# Development of an Interprofessional Training Education Program in Geriatrics

**DOI:** 10.1093/geroni/igaa057.1598

**Published:** 2020-12-16

**Authors:** Sam Cotton, Anna Faul, Pamela Yankeelov, Joe D’Ambrosio

**Affiliations:** 1 University of Louisville, Louisville, Kentucky, United States; 2 University of Louisville Trager Institute, Louisville, Kentucky, United States

## Abstract

This study examines the development of an interprofessional training certificate program that prepares social work learners to infuse geriatrics and behavioral health into primary care settings. Since 2018, our program has trained 31 social work learners and 16 learners from counseling psychology and nursing. At the core of the certificate program is an emphasis on developing skills focused on the integration of geriatrics, behavioral health and primary care to address the lack of workforce trained at the intersection of these areas. Each series of workshop is aligned with core competencies that address the 4-M Model of Age-Friendly Health Care and SAMSHA’s Core Competencies in Behavioral Health. Our professional certificate includes training in Motivational interviewing, as well as Cognitive behavioral therapy, Mindfulness based cognitive therapy and Problem-solving therapy, Narrative Therapy, Strategic Therapy, Systemic Therapy, Life Review and Reminiscence Therapy. Additionally, students receive professionalization trainings to help prepare them for the job market. To measure the efficacy of this curriculum program, we examined the outcomes related to student knowledge of geriatrics and behavioral health including knowledge attainment, fidelity to modalities, learner self-efficacy, and learner satisfaction. The results of this study showed that integrating interprofessional education into social work settings can lead to positive outcomes for student knowledge, self-efficacy and learning satisfaction. Additionally, we found that having a curriculum that focused on interprofessional teams contributed to higher self-efficacy in completing tasks compared to previous cohorts. This has implications for the way that we conceptualization the use of interprofessional education.

